# Trends in the Epidemiological Aspects and Mortality of Alcoholic Liver Disease in Korea in the Decade Between 2000 and 2009

**DOI:** 10.14740/jocmr1975e

**Published:** 2014-11-19

**Authors:** Hyung-Ae Bang, Young-Hwan Kwon, Myeong-Jin Lee, Won-Chang Lee

**Affiliations:** a College of Health Sciences, Korea University, Seoul 136-703, Korea; Korea Public Health Association, Seoul, Korea; b Department of Internal Medicine, Aeromedical Center, Korean Air, Seoul 157-712, Korea; c Public Health in Department of Nutritional Sciences, Otemae College of Nutrition, Osaka 540-0008, Japan; d College of Veterinary Medicine, Konkuk University, Seoul 143-701, Korea

**Keywords:** Epidemiology, ALD, Case-fatality, Mortality rate, ICD

## Abstract

**Background:**

Alcohol consumption and related alcohol liver disease (ALD) have substantially increased in Korea during the last decade. The objective of this study was to evaluate the trends in the epidemiological aspects and mortality rate (MR) of Korea in the decade between 2000 and 2009.

**Methods:**

The raw data analyzed in this study were obtained from the website of “the ALD” managed by Korea Center for Disease Control and Prevention (KCDC), Korea Public Health Association (KPHA), and statistics website of Statistics Korea. The data analyses were performed using Excel 2007 statistical software (Microsoft Corp., USA).

**Results:**

The amount of alcohol-consumption-per-capita-per-year (ACCY) in Korea was 8.38 L in 2000 and 8.54 L in 2009. The most taken alcoholic beverage was soju, followed by beer. There were a total of 1,403 case-fatalities (CF) with an MR of 2.98 per 100,000 populations of ALD in 2000, while a total of 3,588 CF with an MR of 7.21 in 2009 (P < 0.01). The CF and MR of ALD in males were significantly higher than those in females (P < 0.01). In over 40-year-old age groups, the CF and MR were significantly increased (P < 0.01). Moreover, occupational classification revealed that the mistress/students/jobless (MSJ) were the most risky group. The comparison of overall CF and MR of ALD by six key classifications (International Classification of Diseases (ICD)) showed that alcoholic cirrhosis (229 CF and 16.3%) in 2000 tended to be increased in 2009 (2,803 CF and 78.1%), while alcoholic fibrosis and sclerosis (607 CF and 43.3%) in 2000 significantly decreased in 2009 (120 CF and 3.3%), respectively.

**Conclusion:**

ALD is one of the most severe diseases in Korea, as indicated by its high CF and MR in this study. As over-consumption of alcoholic beverages is relatively common in Korea, more efforts should be made toward prevention of ALD by raising awareness of the risk factors of ALD by public health education.

## Introduction

Alcoholic liver disease (ALD) related to alcohol is a very common global problem for public health and is one of the major medical complications of alcohol abuse [1-3]. Moreover, the most enduring insights into the effects of alcohol assert that heavy alcohol consumption increases mortality rates (MRs), especially those from liver cirrhosis and other forms of ALD [4]. In an assessment by the World Health Organization (WHO) in 2005, 4% of the burden of diseases and 3.2% of all deaths globally were attributable to alcohol. ALD is the foremost health risk in developing countries and ranks third in developed countries [5]. Per capita alcohol consumption has declined in the United States, Europe, and Japan in East Asia, except in some northern Europe countries such as the UK and Finland [2, 6, 7]. In a case of Japan, mean per capita consumption of alcohol for Japanese adults has been gradually decreasing for more than 15 years, while it still remains at a high level [7].

In Korea, where alcohol is one of the leading causes of chronic liver disease, 73% of adult Korean men and 36.2% of women drink alcohol more than once a month [4, 7, 8]. Moreover, the quantity of alcohol consumption increased from 8.38 L/capita in 2000 to 8.54 L/capita in 2009 [9, 10]. Additionally, Korean people really enjoy drinking. Although per capita alcohol consumption has tended to increased for more than 10 years, it has always remained at a high level. Diversification of the drinking population has progressed rapidly, specifically in women, among whom alcohol consumption has increased sharply [3, 5]. Consuming large quantity of alcoholic beverages over a long period of time is the major cause of ALD, which encompasses fatty liver, alcoholic hepatitis and chronic hepatitis with hepatic fibrosis or cirrhosis [1-9].

In the present study, we investigated epidemiological aspects and MR of ALD in Korea in the decade between 2000 and 2009, in order to stimulate future strategies for improving public health.

## Materials and Methods

In order to analyze the epidemiological aspects and MRs among patients of ALD in Korea in the years 2000 and 2009, we used raw data of “ALD” obtained from the Statistical System of Korea Center for Disease Control and Prevention (KCDC) [8] and website of Alcohol Statistical System of the Korea Public Health Association [9]. Additionally, the raw data of alcohol consumption were obtained from the Statistics Korea (yearbook) [10].

In this study, the MR of ALDs in patients per 100,000 populations was estimated using the criteria established by the WHO, and the upper and lower limits of the 95% confidence intervals (CIs) were calculated. Statistically significant differences between the epidemiological aspects and risk factors were determined using the Chi-square test or paired *t*-test, and the data analyses were performed using Excel 2007 statistical software (Microsoft Corp., Redmond, WA, USA). Statistically significant levels were at P < 0.05 and P < 0.01.

## Results

Alcohol has been consumed since ancient time in Korea. Through history, the drink of alcoholic beverages has played an important role in social and cultural events in many societies. Comparative observation of trends in alcohol-consumption-per-capita-per-year (ACCY) in Korea in the decade between 2000 and 2009 was analyzed and shown in Table 1. The amount of ACCY in Korea was 8.38 L in 2000 and 8.54 L in 2009. In addition, soju, which showed 5.25 L or 62.7% of the total ACCY in 2000 and 51.5 L or 60.1% in 2009, and 1.92 L or 22.9% in 2000 and 2.11 L or 24.7% in 2009, was more likely to be associated with high risk of drinking than other alcoholic beverages such as makgeolli/takju (0.5 L or 5.9% in 2000 and 0.74 L or 8.7% in 2009) (P < 0.01). Overall ACCY was slightly increased in 2009 compared to 2000, and makgeolli/takju (+2.8%), beer (+1.8%), and wine (+1.5%) were such cases. However, the consumption of soju (-2.6%), cheongju (-0.4%), whisky (-2.4%) and others (-0.8%) were significantly decreased in 2009 (P < 0.05 or P < 0.01).

**Table 1:** Comparisons of Trends in Alcohol-Consumption/Capita/Year (ACCY) in Korea in the Decade Between 2000 and 2009

Item	2000		2009		Changes rate (%)
	Quant. (%)	95% CI	Quant. (%)	95% CI	
Makgeolli/takju	0.50 (5.9)	4.3 - 7.5	0.74 (8.7)	6.8 - 10.6	+2.8**
Soju	5.25 (62.7)	59.4 - 66.0	5.13 (60.1)	56.8 - 63.4	-2.6**
Cheongju	0.11 (1.3)	0.5 - 2.1	0.08 (0.9)	0.3 - 1.5	-0.4*
Beer	1.92 (22.9)	20.1 - 25.7	2.11 (24.7)	21.9 - 27.5	+1.8**
Wine	0.02 (0.3)	-	0.16 (1.8)	0.9 - 2.7	+1.5**
Spirits/whisky	0.44 (5.3)	3.8 - 6.8	0.25 (2.9)	1.8 - 4.0	-2.4**
Others	0.14 (1.7)	0.8 - 2.6	0.08 (0.9)	0.3 - 1.5	-0.8**
Total	8.38		8.54		
P-value	< 0.01		< 0.01		

Quant.: Quantity (unit: L). 95% CI: confidence interval of 95% of the rate. Statistically significant levels were set at *P < 0.05 and **P < 0.01.

In Table 2, the trends of cases and MR of ALD in 2000 and 2009 were compared by nationwide, gender and age groups. There were a total of 1,403 nationwide deaths with an MR of 2.98 per 100,000 populations in 2000, and a total of 3,588 nationwide deaths with an MR of 7.29 in 2009; the MR of ALD increased by more than two times in 2009 (P < 0.01). In addition, men had significantly higher MR of ALD in both 2000 (5.64 per 100,000) and 2009 (13.6) than women in both 2000 (0.31 per 100,000) and 2009 (1.42), respectively (P < 0.01).

**Table 2:** Tends in the Mortality Rate of ALD in Korea Between 2000 and 2009

Item	2000		2009		Changes
	Cases	MR	Cases	MR	
Nationwide					
Mortality rate	1,403	2.98	3,588	7.21	+4.23**
Gender					
Male	1,334	5.64	3,245	13.16	+7.52**
Female	69	0.31	343	1.42	+1.11**
Total	1,403		3,588		
P-value	< 0.01		< 0.01		
Age					
20 - 29	12	0.28	6	0.15	0.13
30 - 39	152	3.49	198	4.76	+1.27**
40 - 49	468	13.26	1,076	31.53	+18.27**
50 - 59	369	16.97	1,230	56.01	+39.04**
60 - 69	259	17.89	721	41.20	+23.31**
> 70	143	20.31	357	27.29	+6.98**
Total	1,403		2,588		
P-value	< 0.01		< 0.01		

MR: mortality rate per 100,000 populations. Statistically significant levels were set at *P < 0.05 and **P < 0.01.

The distribution of the MR by age groups in 2000 was as follows: in the age groups of 20 - 29, 30 - 39, 40 - 49, 50 - 59, 60 - 69 and over 70 years, the MRs of ALD were 0.28, 3.49, 13.26, 16.97, 17.89, and 20.31 per 100,000 populations, respectively, and the MR of ALD by age groups in 2009 was similar to those in 2000. However, MR of ALD in all age groups tended to be increased from 2000 to 2009 (P < 0.01). Moreover, the MR of ALD was the highest in the age group of 40 - 50, and clearly showed higher occurrence in both 2000 and 2009.

A trend in the epidemiological aspects of the case-fatalities (CF) with ALDs in Korea was shown in Table 3. In Korea, men had a statistically significantly higher CF of ALD in both 2000 (1,334 cases or 95.1%) and 2009 (3,245 cases or 90.4%) than women in both 2000 (69 cases or 4.9%) and 2009 (343 cases or 10.5%), respectively (P < 0.01).

**Table 3:** Trends in Epidemiological Aspects of the Case-Fatalities With Alcohol Liver Disease (ALD) in Korea Between 2000 and 2009

Item	2000		2009		Changes
	Cases	% (95% CI)	Cases	% (95% CI)	
Gender					
Male	1,334	95.1 (94.0 - 96.2)	3,245	90.4 (89.4 - 91.4)	-4.7**
Female	69	4.9 (3.8 - 6.0)	343	10.5 (9.5 - 11.5)	+5.6**
Total	1,403		3,588		
P-value	< 0.01		< 0.01		
Age distribution					
20 - 39	164	11.7 (10.0 - 13.4)	204	5.7 (4.9 - 6.5)	-6.0*
40 - 59	837	59.7 (57.1 - 62.3)	2,306	64.3 (63.0 - 65.6)	+4.6**
> 60	402	28.6 (26.3 - 28.9)	1,078	30.0 (28.8 - 31.2)	+1.4**
Total	1,403		3,588		
P-value	< 0.01		< 0.01		
Occupation					
White-collar jobs	167	11.9 (10.2 - 13.6)	408	11.4 (10.4 - 12.4)	-0.5
Blue-collar jobs	159	11.3 (9.6 - 13.0)	279	7.8 (6.9 - 8.7)	-3.5**
Farmer and fishery	282	20.1 (18.0 - 22.2)	409	11.4 (10.4 - 12.4)	-8.7**
Mist/Stud/jobless	780	55.6 (53.0 - 58.2)	2,398	66.8 (65.3 - 68.3)	+11.2**
The other jobs	15	1.1 (0.6 - 1.6)	94	2.6 (2.1 - 3.1)	+1.5**
Total	1,403		3,588		
P-value	< 0.01		< 0.01		

95% CI: confidence interval of 95%. Mist: Mistresses, Stud: Students. Statistically significant levels were set at *P < 0.05 and **P < 0.01.

The proportionality of the CF by age groups in 2000 was as follows: in the age groups of 20 - 39, 40 - 59 and over 60 years, the percentages in 2000 were 11.7%, 59.7% and 28.6%, respectively (P < 0.01), and those in 2009 were 5.7%, 64.3% and 30.0%, respectively (P < 0.01). Particularly, the age group of 40 - 50 showed the highest CF of ALD in both 2000 and 2009.

The proportional distribution of the CF of ALD by occupation in 2000 was as follows: mistress/students/jobless (MSJ), farmer and fishery, white-collar jobs, blue-collar jobs, and the other jobs, the percentages were 56.6%, 20.1%, 11.9%, 11.3%, and 1.1%, respectively (P < 0.01), while in 2009, those percentages were 66.8%, 11.4%, 11.4%, 7.8%, and 2.6%, respectively (P < 0.01).

**Figure 1:**
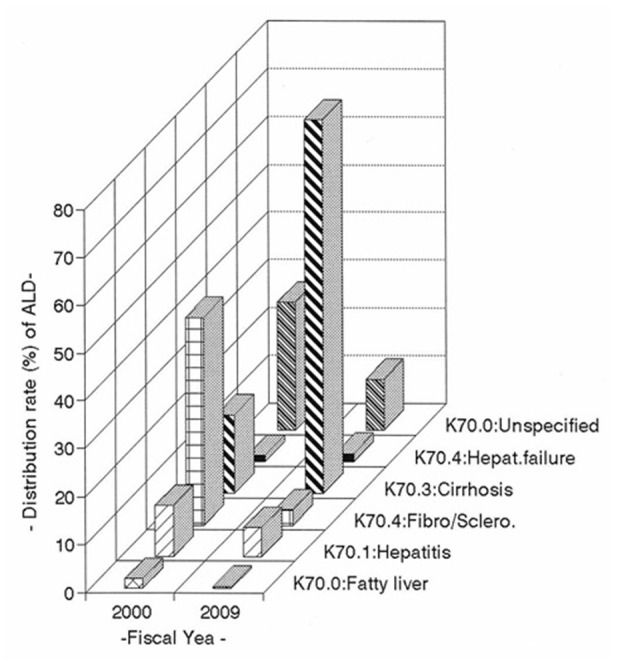
Trends in distribution rate (%) of the case-fatalities of the most prevalent types of alcoholic liver disease by ICD in Korea in the decade between 2000 and 2009.

The trends in the distribution rate (%) of the CF and MR of the most prevalent types of ALD were compared by six key classifications of the International Classification of Diseases (ICD; ICD-10 Version; 2010). As shown in Table 4 and Figure 1, in 2000, a total of 1,403 CF and 2.98 MR of ALD were analyzed: alcoholic fatty liver (1.8% of total CF and 0.05 of MR), alcoholic hepatitis (10.6% and 0.32 MR), alcoholic fibrosis and alcoholic sclerosis (43.3% and 1.30 MR), alcoholic cirrhosis (16.3% and 0.49 MR), alcoholic hepatic failure (1.2% and 0.04 MR) and ALD unspecified (26.8% and 0.78 MR), respectively. In 2009, a total 3,588 CF and 7.29 MR of ALD were reported: alcoholic fatty liver (0.3% of the total cases and 0.02 of MR), alcoholic hepatitis (6.1% and 0.43 MR), alcoholic fibrosis and sclerosis (3.3 and 0.24 MR), alcoholic cirrhosis (78.1% and 5.63), alcoholic hepatic failure (1.6% and 0.12 MR) and ALD unspecified (10.6% and 0.76 MR), respectively (P < 0.01).

**Table 4:** Trends in the Case-Fatalities and Mortality Rate of the Most Prevalent Types of Alcoholic Liver Disease by ICD in Korea Between 2000 and 2009

Item	2000		2009		
Distribution of ALD	Cases	% (95% CI)	Cases	% (95% CI)	Changes
K70.0: Fatty liver	25	1.8 (1.1 - 2.5)	12	0.3 (0.1 - 05)	-1.5*
K70.1: Hepatitis	149	10.6 (9.0 - 12.2)	215	6.1 (5.3 - 6.9)	-4.5**
K70.2: Fibro./sclerosis	607	43.3 (40.7 - 45.9)	120	3.3 (2.7 - 3.9)	-40.0**
K70.3: Cirrhosis	229	16.3 (14.3 - 18.3)	2,803	78.1 (76.7 - 79.5)	+61.8**
K70.4: Hepatic failure	17	1.2 (0.6 - 1.8)	58	1.6 (1.2 - 2.0)	0.4
K70.9: Unspecified	376	26.8 (24.5 - 29.1)	380	10.6 (9.6 - 11.6)	-16.2**
Total	1,403	100.0	3,588	100.0	
P-value	< 0.01		< 0.01		
Mortality rate	Cases	MR (95% CI)	Cases	MR (95% CI)	Changes
K70.0: Fatty liver	25	0.05 (0.02 - 0.08)	12	0.02 (0.01 - 0.03)	-0.03
K70.1: Hepatitis	149	0.32 (0.27 - 0.37)	215	0.43 (0.40 - 0.46)	+0.11*
K70.2: Fibro./sclerosis	607	1.30 (1.20 - 1.40)	120	0.24 (022 - 0.26)	+1.06**
K70.3: Cirrhosis	229	0.49 (0.43 - 0.86)	2,803	5.63 (5.52 - 5.74)	+5.14**
K70.4: Hepatic failure	17	0.04 (0.02 - 0.06)	58	0.12 (0.11 - 0.14)	0.08
K70.9: Unspecified	376	0.78 (0.70 - 0.86)	380	0.76 (0.72 - 0.80)	-0.02
Total	1,403	2.98	3,588	7.21	
P-value	< 0.01		< 0.01		

MR: mortality rate per 100,000 populations. 95% CI: confidence interval of 95%. Statistically significant levels were set at *P < 0.05 and **P < 0.01.

Table 4 also shows the changes of CF and MR between 2000 and 2009; alcoholic cirrhosis (+61.8% and +5.14MR), alcoholic hepatic failure (+0.4% and +0.08 MR) were increased, while alcoholic fatty liver (-1.5% and -0.03 MR), alcoholic hepatitis (-4.5% and +0.11MR), alcoholic fibrosis and sclerosis (-40% and -1.06 MR) and ALD unspecified (-16.2% and -0.02 MR) were decreased.

## Discussion

Korean culture has a great variety of traditional alcoholic beverages. The major crop has historically been rice, and thus most Korean traditional alcoholic beverages have been made from rice, of both the glutinous and non-glutinous variety, which are fermented with the aid of yeast and nuruk, a wheat-based source of the enzyme amylase. Moreover, Koreans often use fruits, flowers, herbs, and other ingredients to flavor these beverages, to a much greater extent than Chinese wine. Additionally, in Korea, makgeolli/takju and soju have long been popular alcohol beverages which are made from grains. Particularly, soju is made from grain or sweet potatoes, while it is distilled from rice, barley and a wide range of other grain. It typically has an alcohol content of 40 proofs (20% alcohol by volume) [11, 12]. In Korea, soju (5.13 L of ACCY) and beer (2.11 L) were most common alcoholic beverages as shown in Table 1. In recent years, the increasing availability and accessibility of alcoholic beverages lead to changes in drinking patterns across the globe [1-5, 10, 13]. But alcohol can cause dangers even when one is not dependent on it. Drinking beyond reasonable levels raises the risk of acute alcoholic poisoning, lifestyle-induced illnesses (ALD), hepatic-cancer, depression and other health problems [1-4, 12, 13]. For example, one epidemiological study has estimated that every 1 L increase of alcohol consumption per capita (independent of type of beverage) caused 14% increase in cirrhosis in men and 8% increase in women [14, 15]. Consuming a large quantity of alcoholic beverages over a long period time is a major cause of ALD, a term that encompasses the hepatic manifestation of alcohol over-consumption, including alcoholic fatty liver, alcoholic hepatitis and chronic hepatitis with hepatic fibrosis or cirrhosis [1-9].

In Korea, the total CF and MR of ALD in 2009 were increased by more than two times compared to those in 2000. In addition, the MR of ALD of men was much higher than women. The remarkable differences of MR of ALD in gender are believed to be due to differences between men and women in terms of the differences between the sexes. The tolerance to alcohol of women is less than that of men [1-3, 12, 16]. It is also true that men typically drink more than women, and that the proportion of heavy drinkers and alcoholics is much higher among men. Moreover, the levels of alcohol dehydrogenase, an enzyme involved in breaking down alcohol, may be lower in stomachs of women than men, which would result in a higher blood alcohol content for women than for men who consume equivalent amounts of alcohol [4, 15, 17].

In this study, the percentage distribution of the CF and MR of ALD in 40 - 50 age group was the highest in all age groups between 2000 and 2009. Although ALD will develop in any individual who consumes a large quantity of alcoholic beverages over a long period of time, this process is transient and reversible [1-2, 12, 18, 19].

Occupational mortality analysis presents a number of challenges, including the bias that can occur because of differences in the occupation recorded at death and census [8, 9]. Among those who drink, self-employment or MSJ were positively associated with consumption levels, and white- and blue-collar jobs were negatively associated. The most prevalent types of alcohol-induced ALD were alcoholic fatty liver, alcoholic hepatitis, and cirrhosis. Often, as people continue to drink heavily, they progress from fatty liver to hepatitis to cirrhosis [1-4].

ALD is a major cause of alcohol-related morbidity and mortality [1-4]. Although the amount and pattern of alcohol consumption is well-recognized predisposing factor for the development of serious liver pathology, environmental factors and the host’s genetic make-up may also play significant roles that have not yet been entirely explored [1, 4, 15]. However, in a practice, most patients with alcoholic hepatitis in ALD drink more than 100 g/day (which corresponds to 6 - 7 drinks per day where one drink contains 13 - 15 g of alcohol), with 150 - 200 g per day being common [18-20]. In addition, liver-related from alcohol contributes to 4% of mortality and 5% disability adjust life years (DALY) globally, with highest impact in Europe [21, 22]. The most prevalent type of ALD is fatty liver, alcoholic hepatitis, and cirrhosis. Often, as people continue to drink heavily, they progress from fatty liver or simple steatosis to hepatitis to cirrhosis [4, 15, 18]. For example, fatty liver develops in about 90% of individuals who drink more than 60 g/day of alcohol, but may also occur in individuals who drink less [15, 23]. However, several studies have suggested that progression to fibrosis and cirrhosis occurs in 5-15% of patients despite abstinence [15]. Fibrosis is believed to start in the perivenular area and is influenced by the amount of alcohol ingested [15, 23]. Perivenular fibrosis and deposition of fibronectin occur in 40-60% of patients who ingest more than 40 - 80 g/daily for an average of 25 years [18, 24]. In addition, cirrhosis of the liver disease is the most serious form of ALD and a cause of many death and illnesses in Korea [8-10]. According to previous report, about 10-15% of people with alcoholism develop cirrhosis, but many survive it. Many are unaware that they have it, and about 30-40% of cirrhosis cases are discovered at autopsy [4, 25].

In the present study, it was obvious that alcoholic cirrhosis was increased significantly in 2009 (2,803 CF and 78.1%) compared to 2000 (229 CF and 16.3%). ALD is a highly lethal disease and is associated with the consumption of the alcoholic beverages [1-9]. Therefore, control measures should focus on the increased risk of alcoholism, and recognize the present state of alcohol consumption, and then more efforts must be made to reduce the consumption of alcoholic beverages for public health.

In conclusion, as ALD is one of the most severe and relatively common diseases in Korea, more efforts should be made towards prevention through raising awareness of the risk of consumption of alcoholic beverages. Because of its rapid aggravation and increasing prevalence with high CF and MR, public health education about alcohol consumption is strongly recommended for avoiding ALD. In the present study, we provided useful quantitative cross-section analysis of trends in the epidemiological aspects and MR of ALD in Korea. It is hoped that this information would be a useful reference for the further studies of ALD in the field of public health service.
